# Face Masks During the COVID-19 Pandemic: A Simple Protection Tool With Many Meanings

**DOI:** 10.3389/fpubh.2020.606635

**Published:** 2021-01-13

**Authors:** Lucia Martinelli, Vanja Kopilaš, Matjaž Vidmar, Ciara Heavin, Helena Machado, Zoran Todorović, Norbert Buzas, Mirjam Pot, Barbara Prainsack, Srećko Gajović

**Affiliations:** ^1^MUSE – Science Museum, Trento, Italy; ^2^Faculty of Croatian Studies, University of Zagreb, Zagreb, Croatia; ^3^Croatian Institute for Brain Research, University of Zagreb School of Medicine, Zagreb, Croatia; ^4^Institute for the Study of Science, Technology and Innovation, The University of Edinburgh, Edinburgh, United Kingdom; ^5^Business Information Systems, Cork University Business School, University College Cork, Cork, Ireland; ^6^Communication and Society Research Centre, University of Minho, Braga, Portugal; ^7^University Hospital Medical Center “Bežanijska kosa”, and University of Belgrade Faculty of Medicine, Belgrade, Serbia; ^8^Department of Health Economics, Faculty of Medicine, University of Szeged, Szeged, Hungary; ^9^Department of Political Science, Centre for the Study of Contemporary Solidarity (CeSCoS), University of Vienna, Vienna, Austria; ^10^Department of Global Health & Social Medicine, King's College London, London, United Kingdom

**Keywords:** COVID-19, face mask, physical distancing, health communication, personal protecting equipment

## Abstract

Wearing face masks is recommended as part of personal protective equipment and as a public health measure to prevent the spread of coronavirus disease 2019 (COVID-19) pandemic. Their use, however, is deeply connected to social and cultural practices and has acquired a variety of personal and social meanings. This article aims to identify the diversity of sociocultural, ethical, and political meanings attributed to face masks, how they might impact public health policies, and how they should be considered in health communication. In May 2020, we involved 29 experts of an interdisciplinary research network on health and society to provide their testimonies on the use of face masks in 20 European and 2 Asian countries (China and South Korea). They reflected on regulations in the corresponding jurisdictions as well as the personal and social aspects of face mask wearing. We analyzed those testimonies thematically, employing the method of qualitative descriptive analysis. The analysis framed the four dimensions of the societal and personal practices of wearing (or not wearing) face masks: individual perceptions of infection risk, personal interpretations of responsibility and solidarity, cultural traditions and religious imprinting, and the need of expressing self-identity. Our study points to the importance for an in-depth understanding of the cultural and sociopolitical considerations around the personal and social meaning of mask wearing in different contexts as a necessary prerequisite for the assessment of the effectiveness of face masks as a public health measure. Improving the personal and collective understanding of citizens' behaviors and attitudes appears essential for designing more effective health communications about COVID-19 pandemic or other global crises in the future.

*To wear a face mask or not to wear a face mask?*

*Nowadays, this question has been analogous*

*to the famous line from Shakespeare's Hamlet:*

*“To be or not to be, that is the question.”*

*This is a bit allegorical*,

*but certainly not far from the current circumstances*

*where a deadly virus is spreading amongst us*... Vanja Kopilaš, Croatia.

## Introduction

The coronavirus disease 2019 (COVID-19) pandemic is currently perceived as one of the greatest global threats, not only to public health and well-being, but also to global economic and social stability. While the first two decades of the third millennium were characterized by crisis—most notably the economic downturn of 2008 and the looming climate change—the spread of the severe acute respiratory syndrome coronavirus 2 (SARS-CoV-2) virus originating from China has given rise to most drastic societal and political responses. These included measures as severe as states forbidding citizens from leaving their homes and effectively shutting down all social and economic activities (1). In Europe, Italy was the first country to officially detect the presence of COVID-19 in its territory, and it swiftly adopted measures to contain its spread (2–4). Within a few weeks, the epidemic progressively spread across Europe. Because of the novel situation and the contradictory opinions of experts, including representatives of the scientific community and World Health Organization (WHO), the level of threat caused by the disease appeared unclear (5). The assessment of the perceived risks of the disease varied in the public discourse—some considered it just as “a stronger influenza”; others drew parallels with the very deadly Spanish Flu outbreak in the 1918–1920, and many were simply not sure what to believe. Nevertheless, most felt the novel and unpleasant feeling of being vulnerable to the invisible threat of the infection (i.e., to be the ones in danger) or to be contagious themselves (i.e., to be the danger).

A variety of public health and hygiene measures have been initiated; the most visually noticeable perhaps is the wearing of face masks. The medical research on the use of face masks as personal protective equipment (PPE) against SARS-CoV-2 transmission was interpreted very cautiously, and the initial guidance from health officials was conflicting (6). The WHO advice was conceived to avoid unnecessary paternalism and at the same time be comprehensive in discussing different medical aspects of mask use. However, it was updated several times, shifting from initial statements that face masks are not to be worn by healthy individuals toward gradual adoption of face masks as useful in slowing community transmission. In particular, “…WHO has updated its guidance to advise that to prevent COVID-19 transmission effectively in areas of community transmission, governments should encourage the general public to wear masks in specific situations and settings as part of a comprehensive approach to suppress SARS-CoV-2 transmission” (7). Gradually, face mask use has been recognized as a suitable measure within the scientific community (8–12), if nothing else due to the application of the “precautionary principle” in the face of an acute crisis (13, 14). This has since been backed up by empirical observations (15, 16).

Different, mandatory or voluntary, practices, and contradictory indications about the utility of face mask wearing were introduced across affected countries. Generally speaking, face masks have been adopted as one of the measures to reduce the COVID-19 spread across Europe, despite the fact that wearing masks in Europe is not common or familiar, and it is often associated with Asian countries (17). The social conventions and personal meanings of face mask use have received relatively little attention. Its use is deeply connected to social and cultural practices, as well as political, ethical, and health-related concerns, personal, and social meanings (18, 19).

In this study, our aim was to address three aspects of face mask wearing—public policies, individual behaviors and attitudes, and the collective experiences of the affected communities. In order to develop insights into the wider meanings of face mask wearing beyond (just) preventing the spread of infection, we tapped into the expertise of a scholarly interdisciplinary network, the Navigating Knowledge Landscapes—NKL (http://knowledge-landscapes.hiim.hr/), predominantly consisting of Europe-based scholars. The network is dedicated to furthering research on topics related to medicine, health, and society and comprises academics working across the disciplinary spectrum. We invited NKL members in May 2020 to provide their observations on the topic, also based on their professional experience. They were asked to describe the face mask usage in their countries and provide their subjective standpoints and/or those from their social environment. Subsequently, these testimonies within the specific time window (May 2020) containing narratives on face masks from the contributing experts were thematically analyzed using the method of qualitative descriptive analysis (20, 21).

## Materials and Methods

The invitation to write their views about face mask wearing was sent by e-mail to 97 experts, all members of the interdisciplinary research network Navigating Knowledge Landscapes (NKL; http://knowledge-landscapes.hiim.hr/). The invitation was sent on May 11, 2020, and the responses were collected until May 26, 2020 (over 16 days' period). The experts were asked to contribute a single-page narrative structured in four parts, framed as follows:

- Part 1: What are the rules adopted in your country about face mask wearing? What would be the overall approach for use of the face masks in your community (government instructions, availability, the citizen compliance)?
- Part 2: What is your individual/personal attitude and practice in relation to face masks? If applicable, start with good practice and end with what you consider to be mistakes.
- Part 3: How do you judge the behavior of people you encounter? Face masks (or no face masks) and interpersonal interactions. Again, start with positive and end with negative.
- Part 4 (optional): free to say whatever you think is important to the practices of your community in relation to face masks.

Twenty-nine scholars responded (30% of those invited), providing 27 contributions (two contributions were coauthored). They were from 22 countries, 20 from Europe (Albania, Austria, Bosnia and Herzegovina, Croatia, Czechia, Estonia, Hungary, Italy, Ireland, Norway, Poland, Portugal, Romania, Serbia, Slovenia, Spain, Sweden, Turkey, Ukraine, and United Kingdom) and two from Asia (China and South Korea). The contributors belonged to the following academic disciplines: biology (2), economics (1), engineering (2), information systems (1), law (1), medicine (6), philosophy (5), psychology (1), and sociology (10).

The contributors as experts are all highly educated (Ph.D., holders or Ph.D., students), and most of them are employed in academic institutions and perform research activities in their respective disciplines. The authors of this study were among the contributors.

The testimonials were based on the aforementioned open-ended questions and narrative in style. “Face mask” was used as the umbrella term for all types of face coverings, from the custom-made cotton scarves to disposable surgical masks and medical-grade N95 respirators. This was done to preserve the authenticity of these narratives without going into detail about the medical or microbiological features of the different types of face coverings. In the same way, grammatical or vocabulary use of non-native English speakers was kept as it was. The contributions received were collected and published as a citable open-source dataset at Mendeley Data repository (22).

The contributions were thematically analyzed by employing a qualitative descriptive approach (23). We chose this method because it aimed to provide “rich descriptions about a phenomenon, which little may be known about” [(23), p. 3] and was particularly useful for exploratory research such as our study. It is characterized by staying close to the empirical data, instead of seeking to provide a more conceptual interpretation of the phenomenon in question. Moreover, open-ended questions address different aspects of the same topic and allow formulating answers that could let respondents to frame face mask wearing according to their own personal views (24).

Concerning the thematic analysis, we divided testimonials in three categories. The first category captured the situation in the respondent's country; the subcategories we were interested in were the regulatory framework and the supply situation in each respective country. The second category captured experts' own use of masks. Here we focused in particular on whether and in which situations they reported to wear (or not wear) masks, what kind of face covering they used, and the meaning they ascribed to masks (e.g., mask wearing as a symbol of social cohesion). Third, we categorized the participants' accounts regarding the practices and attitudes of mask wearing they observed in others. We created an MS Excel file in which we collected the respondents' statements on these different categories. In a subsequent step, we analyzed the data for patterns and recurring topics. We looked for country-specific differences and similarities in regulations and practices. Moreover, we also paid close attention to how the experts made sense of their experiences with mask wearing and how the issues addressed were expressed (e.g., experts referring to folk stories, metaphors, or past incidents). When presenting our research results, we focused on the topics we identified as prevalent through our inductive analysis, and we contextualized it based on the published research.

### Ethics

The narratives analyzed in this study were given with the full consent of the people who wrote them and were made available for public access as an open-source repository for the research purpose (22). All the authors provided their consent that the narratives are published in the repository under their full name and affiliation and that they can be used for research purposes. The authors were cited here under their full names, recognizing their authorship of the narratives and their contribution to the dataset collection. The study received ethical approvals from the Ethical Committees of the University of Edinburgh, Scotland, UK and the University of Zagreb, Faculty of Croatian Studies, Croatia.

## Results

### Face Mask Wearing From Medical to Public Settings

The use of a face mask—of various specifications according to the required degree of protection/function—is part of the PPE required in several professional activities, most noticeable in healthcare. One of the participants in this study, who works in healthcare, described her own experience in terms of the caring features of the face masks from medical to communal setting.

“*As an obstetrician–gynecologist, I am used with the mask, I feel it a part of my professional life, and I am trying to convince people that there is no way of considering the mask as an enemy but as a protection-like and umbrella against the rain, like a coat against the cold—and as a sign of civilization to protect our colleges and people around.” [Iuliana Ceausu, Romania]*

The contextual transfer of face mask use from healthcare settings to public spaces is precisely the aspect of making the “outside world” closely resemble scientific apparatus. This includes measuring its success as a feature of the social power derived from the accuracy of the scientific prediction. For instance, Latour (25) specifically examines the public nature of Pasteur's demonstration of the efficacy of the process of animal vaccination by making a “prophecy” that vaccinated cattle on a pilot farm will survive, while other infected animals will perish. In the same way, the (anecdotally) apparent success of the use of face masks reinforces the belief in their utility and efficacy:

“*The people working in the shops would use the masks too… I see familiar faces of the employees all the times of lockdown, although they spend all time in the shop with many different customers, obviously they did not get sick. This was for me a major reassuring fact that the danger is not so high as it could be seen from the media.” [Srećko Gajović, Croatia]*

It is worth remembering here the significant number of deaths of inadequately protected healthcare workers during the COVID-19 epidemic in various countries, mainly due to the lack of the appropriate PPE supplies (26).

### The Politics of a Face Mask

Following initial confusion around the utility of face masks for slowing down the spread of COVID-19 pandemic, there is increasing scientific evidence to support citizens' wearing of face coverings, albeit the public health advice and legislation vary from country to country. A recent study in Germany indicated that a mandatory approach to face mask wearing achieved better compliance than voluntary one, and it was perceived as an effective, fair, and socially responsible measure (27).

In our study, accordingly, the reported country policies differed across rather a wide spectrum of approaches—ranging from legally mandated instructions to cover one's face in all public spaces reinforced by financial penalties (i.e., payable fines), to recommendations only, official indifference, or advice against this practice (Table 1). We were interested how these policies related to the concurrent COVID-19 situation expressed as total number and increase of cases per million people in these countries during the period when experts made their contributions. We observed an obvious trend showing that the countries with more strict rules had better epidemiological situation than those not mandating the face mask usage (Table 1).

**Table 1:** Perception of the official policies on face mask usage in May 2020.

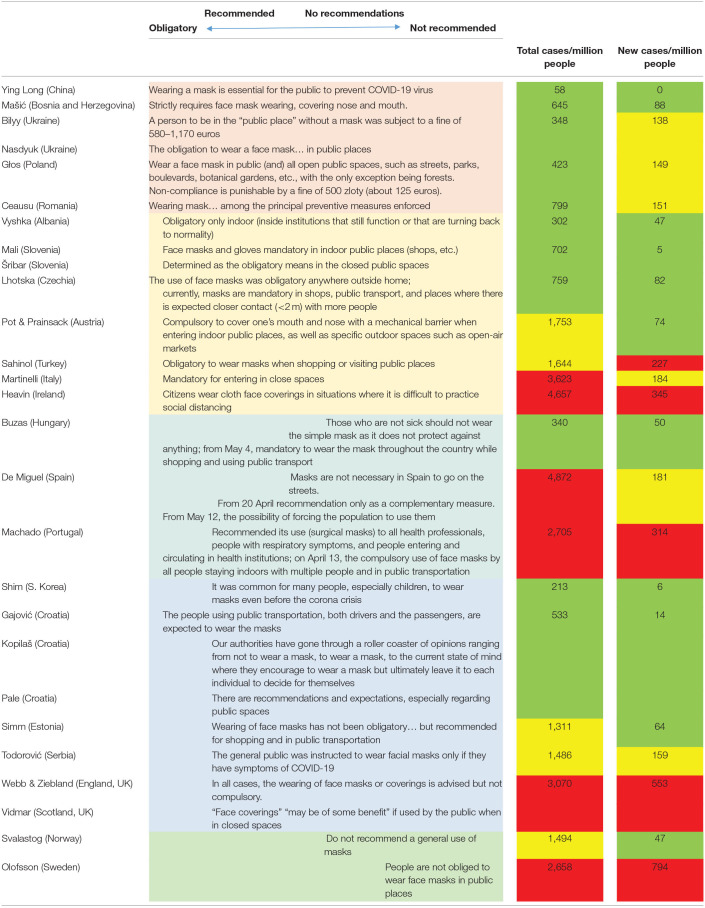

*The official policies (in May 2020) on face mask wearing expressed by the participants in this study in their respective countries. They were distributed across the wide spectrum from mandatory connected with fines, to no recommendations to do so*.

*The experts' perceptions in the first two columns were associated to the numbers in the last two columns representing total cases per million people at the start of the study (May 11, 2020) and new cases per million people during the narrative collection period (May 11–26, 2020) in the corresponding countries (28). The numbers clustered as (green) <1,000 total or <100 new cases per million people, (yellow) between 1,000 and 2,000 total or 100 and 200 new cases per million people, and (red) more than 2,000 total and 200 new cases per million people*.

*It should be noted that the Table concentrated on the time period of the study as official advices, legislation, and numbers of cases subsequently changed during the course of pandemic*.

In some countries, face mask–related policies did not need to be prescribed as this was part of existing established habits; in the same way, no fines are necessary to get people to wash their hands. In particular, since the SARS epidemic in 2003, in many Asian countries, masks are customary wear used to protect against seasonal flu and the common cold. In China and South Korea, they are also employed to protect citizens from pollutants (17, 29).

“*In South Korea, it is common to wear a mask to keep the cold from getting worse in the winter and to prevent the spread of cold to others. Also, as the yellow dust from China and fine dust became much severe, it was common for many people, especially children, to wear masks even before the corona crisis. For this reason, many families even had a lot of masks in their homes before the corona crisis. Personally, I'm familiar with wearing a mask, and I'd like to wear it in order not to harm other people, as I may be a potential patient.” [Jiwon Shim, South Korea]*

In contrast, in the West, the use of face masks is rare in social settings. Hence, because of the public visibility of face mask usage, face masks became an ideological symbol in some countries, with divergent political mindsets governing their adaption or rejection (17). Political dividing lines were particularly apparent in the United States, where the President refused to wear a mask until the last days of July 2020, when the floundering poll numbers and the increasing numbers of COVID-19 cases prompted the need to recommend this health protection device (30). Thus, in the United States and elsewhere, face masks were used by citizens to express their opinions in public.

“*At the beginning of the pandemic, the use of masks had political connotations: since the government advised against their use, their wearing was even considered a form of political opinion.” [Iñigo de Miguel Beriain, Spain]*

The public statement made by wearing (or not wearing) the face mask did not only address the political standpoints but have also been used to communicate various societally relevant statements, i.e., stating ethnical, religious, or cultural affiliations (31). For instance, many countries that before COVID-19 banned face coverings in public spaces are now mandating it, supporting the idea that the past bans were motivated on the basis of religious/cultural beliefs (17).

“*Ethical and moral dilemmas have already risen, especially in countries where Muslim minorities live. If you ban a burka covering the face due to security reasons, how would you deal with massive usage of face masks?” [Gentian Vyshka, Albania]*

“*The decision to wear a face mask is not an easy one. Traditionally, face coverings are an indicator of political persuasion and religious belief. I perceive that the widespread covering of one's face in public is a significant cultural and social shift in Ireland.” [Ciara Heavin, Ireland]*

### “To Wear a Face Mask, or Not to Wear a Face Mask, That Is the Question…”

The collected narratives indicated that the contributors had a clear standpoint on their own face mask usage and developed arguments to support their decisions to wear or not to wear face masks.

“*As soon as I leave the house and find myself in the supermarket or in public places, I wear a mask. However, I do not wear a mask when I take a walk in the forest. I started wearing it even before it became mandatory. I think it is important to wear masks, especially to avoid endangering others, e.g., elderly people. I find it unspeakable when people who wear masks are ridiculed by those who do not wear masks. At least that's what happened to me in the beginning, before the mask duty… Many thought that the people wearing masks would want to protect themselves in particular. Very few thought that people wearing masks wanted to protect their social environment.” [Melike Sahinol, Turkey]*

“*My personal view is that as long as the spread of the virus is under control (as it currently is), there is no need to make the masks obligatory. I personally have not worn a mask (have not purchased any either) with the exception of when I visited healthcare institution (provided by them). I must also say, though, that none of my family members are considered a vulnerable population. If my grandmother would live with us, I might think differently.” [Kadri Simm, Estonia]*

What was exemplified in many narratives is that individual usage is not meant predominantly for an individual's self-protection, but the decision was based on people's relationship to others. The citizens' question “should I protect myself” evolved into “can I protect the others?”

“*I wear disposable masks, understanding they protect others from me, more than me from others. I wear them to demonstrate responsible behavior and attitude to benefit of society.” [Predrag Pale, Croatia]*

The experiences of interaction with others in relation to face mask wearing were mentioned frequently, indicating the importance of the social context of individual behavior.

“*I experienced cases when my request to keep distance or to take on a mask properly was treated offensively or as a sign of mistrust…” [Christina Nasadyuk, Ukraine]*

“*I put it on when I go to the grocery store because at the early stage of the pandemic, I was warned by the lady working at the counter that I am putting her life ‘in danger by not wearing a mask.' Obviously, I did not want to take chances with her life again, so I purchased one of those cloth masks.” [Vanja Kopilaš, Croatia]*

However, many testimonies pointed out that masks have not been used properly. The health risks of incorrectly wearing a face mask represent an important argument against the use of face masks as a public health measure (32).

“*…25% wore masks improperly, on their necks, or covering only their mouths, but not noses. …They do not know how to put the mask on, and when they remove their masks, they touch the outside of the mask, which is inappropriate and wrong.” [Izet Mašić, Bosnia and Herzegovina]*

“*Also, one can observe many cases of half-compliance or sham compliance. For instance, people do wear masks, but slide them down onto their chins or take them off completely while talking to someone on the street or speaking on the phone. And this is all a performance, keeping their masks somewhere within reach in case of the sudden emergence of police officers, who are indeed issuing fines for not wearing a mask.” [Aleksandra Głos, Poland]*

This is even more complicated in situations when face masks were scarce (the stocks gradually improved through time in all examined locales).

“*During the early stages of disease progression, mask wearing was not a common practice, mainly due to the complete absence and highly inflated prices in stores.” [Rostyslav Bilyy, Ukraine]*

“*I do not use face mask. In the early stage of the COVID-19 epidemic in Norway, my understanding was that available masks should be reserved for people in the health and caring sector.” [Anna Lydia Svalastog, Norway]*

“*I think the biggest concern is that the mask has been in short supply for a long time, and that its trade has not been subject to official pricing, so prices have been uncontrolled… The mask was in short supply when emergency was announced, but it is now available in many places and can be obtained at the checkout of almost every grocery store if someone started shopping without it.” [Norbert Buzas, Hungary]*

The shortage of masks ignited a burst of creativity in producing homemade masks, with a proliferation of tutorials for their production on the Internet and social media.

“*Nowhere was possible to come to the face masks. Typical situation: the government did announce decree, but it did not provide the means for its implementation. We as ordinary citizens need to improvise with needlework of masks at home as well. Taking in regard that immediately rapacious war profiteers did appear by selling masks the needlework of masks at home was even not the worst solution.” [Franc Mali, Slovenia]*

“*Although during the first weeks there was lack of masks and respirators, it was great how many people proved their creativity. It concerned not only the textile reusable masks, but also design and development of respirators with higher level of protection. They were mostly printed on 3D printers. Later on, some of the approved types were taken by larger producers, and mass production started.” [Lenka Lhotska, Czechia]*

### Mask Wearing at the Interface of Personal and Social Responsibility

Besides being shaped by public discourse and social norms, risk perception also has a strong personal element. Some people seem like they do not care; others are quite relaxed, and some are more cautious. As for COVID-19, conflicting perspectives and emotions and even the psychological entrapment syndrome known as “cabin fever” (i.e., referencing long winter isolation in a small cabin) have been reported (33). Here, restricted microenvironments and quarantine are felt as secure places. The additional challenges were noticeable during the shift from the lockdown phase and the beginning of the so-called “phase 2” or “reopening” when people were allowed to leave their home again.

“*‘Convivere,' i.e., ‘live together with' the virus is the expression used by experts and media, to describe the phase 2, but this narrative could result quite distressing: how glad would someone be when living with a submicroscopic entity, that is such dangerous?” [Lucia Martinelli, Italy]*

During this second phase, going back to living with “the others” demands new social behavior/etiquette combined with increased safety measures. The face masks start to be part of the new everyday rituals of saying hello, having a coffee together, and protecting each other. The role of peers in shaping the behavior of others is significant. People not committed to wearing mask can feel peers' pressure to comply. Moreover, “a collapse between the status of *being at risk* and *being a risk*” was noted (34–36).

“*The face mask, I realize, signals both positions, at the same time as it doesn't provide a definite answer: are you the risk object or the object at risk? Saying this, my individual attitude toward face masks cannot be pried apart from the social acceptance and use of the same. As long as the nonuse of face masks constitutes the norm, I will most likely interpret the usage as deviant and worrying. On the other hand, if the vast majority of the Swedish population would wear face masks, I would most likely start wearing a face mask as well. Here, the mass effect kicks in.” [Jennie Olofsson, Sweden]*

“*The massive use of the masks among Albanian citizens… has become a normal well-adopted ritual of surviving, implemented as of a social significance for ‘not letting the virus in.' This social cohesion on the intrapersonal view as ‘to scare the virus” and ‘fear of an enemy' comes close to a group approach of ‘control and stability.' This ritual of social cohesion vis-à-vis the ‘fear of death' or ‘fear of the unknowing' is a similar to a psychological regression, when the individual survival depended largely from the herd.” [Gentian Vyshka, Albania]*

“*For me, unlike other measures to contain the spread of the virus, the wearing of masks is predominantly a symbol of social cohesion and complying with the rules and not so much a measure to effectively protect myself and others from infection. The few times I saw someone without a mask entering a supermarket or the metro, my first thoughts were about social deviance and the arrogance of ignoring a commonly agreed-upon practice, and not about the risk of infection.” [Mirjam Pot & Barbara Prainsack, Austria]*

Individual and collective responsibility and trust in the institutions and in the official assessment of risks and recommendations as to the adopted measures are crucial to build up a degree of epistemic agreement (37). However, this is perhaps more challenging in a contested environment of “recommendation trust” (38), which likely depends on communicating certainty (39), of which very little has been seen during COVID-19 pandemic. Hence, the acceptance of official advice varied among countries, cultures, and political contexts, with some degree of contradiction.

“*In general, there seems to be a relatively wide acceptance of government recommendations, but a very patchy uptake. Though the Scottish Government advice is trusted more than that from the UK Government, significant generational and cultural differences can be seen as to its implementation… in a multicultural society such as Scotland, there are some subtle differences between people from different cultural backgrounds and traditions who are either more accustomed to follow stricter government instructions, or from cultures where face mask wearing is more commonplace.” [Matjaž Vidmar, Scotland, UK]*

“*Finally, as an anecdote, I would mention the recent case of expelling an opposition MP from the Assembly because he did not have a mask on his face, although the Prime Minister who warned the MP did not have a mask either.” [Zoran Todorović, Serbia]*

The pandemic also seems to have reminded many people about the responsibility of humanity toward the preservation of all the living organisms and, as recognized by the Centers for Disease Control and Prevention (40), that our health is closely connected to the health of whole environment.

“*We should see ourselves as the most important participants and the biggest beneficiaries of public health, so we should take expert advice—wear mask. In other word, under this special situation, we need to work with medical experts, government to co-build a safe, harmonious and orderly living world with ‘One Health' concept, rather to resist or despise it.” [Bie Ying Long, China]*

### The Face Mask: A New Barrier Affecting Social Relations?

If we assume that in the near future we will be used to living with the pandemic, or even a series of pandemics, we are currently developing new norms for social interaction. Being with other people and enjoying their company are essential for our mental and physical well-being. How do these interactions include face mask usage? What will socializing look like in the era of physical distancing (i.e., “keeping a safe space between yourself and other people who are not from your household”) (41)? These issues are being recognized as particularly challenging.

“*We must reinforce the message that face masks do not remove (or even reduce) the need for social distancing as well as excellent hand and respiratory hygiene. We need to avoid a situation where face masks become a weapon that could negatively impact our fight against this invisible enemy.” [Ciara Heavin, Ireland]*

“*I believe the benefits of face masks may be overestimated and lead us into a false sense of security in which we take unwarranted risks—such as touching more objects and neglecting handwashing or going outside when suffering from a cough or cold. Therefore, my preference would be to give greater attention to other steps such as providing screens and visors for workers in public facing roles and reinforcing protective mechanisms around social distancing.” [Helena Webb & Sue Ziebland, England, UK]*

“*Since the use of a mask started to become widespread, people seem to feel safer and unfortunately are more at risk, for example, not maintaining physical distance, making appointments with extended family and friends, etc.” [Helena Machado, Portugal]*

Not all evidence is in support of above assessments that face masks bring about a (false) sense of security. In a recent study conducted in the Italian Venice metropolitan area, wearing a mask has proven to be a visual factor strengthening physical distancing as a public health measure (3). Between February 24 and April 29, 2020, distances have been measured by an operator wearing an exclusive sensor-based “social distancing belt.” They were interchangeably “unmasked,” “masked,” “do it yourself (DIY)-masked,” “goggles masked,” and “goggles DIY-masked.” Results show that people tended to stay closer to an unmasked person, while mask wearing tended to increase the physical distance. This paradox is explained by considering humans' intrinsic social nature that favors social vs. antisocial behaviors (3). Wearing a mask thus can turn unconscious social behavior into conscious antisocial behavior.

“*I believe that due to the extraordinarity of wearing face coverings in public spaces in Scotland, these do not encourage an undue feeling of ‘safety' by their use, rather the reverse. Hence, with full awareness that the evidence for being protected by this measure is not there, rather, I hope that by wearing a face covering, I may remind (or even deter) others from breaking social distancing rules.” [Matjaž Vidmar, Scotland, UK]*

Marchiori's study (3) also suggests that distance increases with face mask wearing, thus supporting the importance of visual stimuli as a signal of danger. This fact recalled in the mind of our colleague, Bie Ying Long, the ancient Chinese tale of “The Blind Man Who Lights a Lantern While He Walks in the Night,” which proposes a “wise” interpretation of action as interplay of altruism and self-interest (42). When people asked a blind man for the reason why was he carrying a large lantern when he traveled at night, he replied that while day and night were not different to him, carrying a lantern while walking in the night was for the sake of everyone. For him, the lantern provided protection from other people, allowing them to avoid bumping into him. For others, carrying a lantern shone a light on them and let them walk more securely.

“*In the present, we should learn the kind of survival wisdom of the blind man in the story. To wear a mask proactively does not mean ‘I'm infected with the virus,' rather to protect my own health. At the same time, it is a reminder to others that we are still in a time of crisis; we need to pay highly attention to our health and life safety very seriously.” [Bie Ying Long, China]*

However, face mask use may have adverse systemic effects, as well:

“*The use of a mask is seen as an act of responsibility and altruism. However, I notice that people with masks tend to avoid personal interaction and to decrease the time they talk to each other. They avoid looking at others.” [Helena Machado, Portugal]*

“*The syntagm social distancing is problematic because it symbolically transforms the rule of physical distance into the subversion or deconstruing of social ties. Face masks are strongly related to this implicated meaning. The human estrangement as a part of the ‘COVID-19 regime' is the reason I have been more annoyed by some people strongly emphasizing the need for masks and physical distance than by those exhibiting the lack of interest for the personal protection against the infection.” [Renata Šribar, Slovenia]*

In this framework, institutional health communication plays a crucial role in motivating citizens to wear face masks and use them properly (i.e., how to handle it and how to cover one's mouth and nose), as well as to respect physical distancing and hygiene procedures. Here, the choices of narratives by public health system officials play a crucial role. Accordingly, the expression “social distance” tends to be avoided nowadays. “Physical distancing” has been adopted by the WHO, which they define as keeping a distance and avoiding spending time in crowded places or in groups (43). More distressing expressions such as “avoiding all unnecessary contacts” and “unnecessary contacts with the others” are used in some official advices (44). These messages may appear authoritarian, by intruding in the personal space of what is “unnecessary” and about who are “the others” when considering social contacts and human relations.

Conversely, an interesting example for motivating the correct use of face masks is the communication campaign “Per tornare tutti insieme a sorridere” [To get back to smiling together] by the Italian Health Ministry (45). This message designed to stimulate feelings of mutual protection and solidarity among relatives, as well as among strangers. Motivation is crucial because, as we have demonstrated, a face mask can be perceived as both a physical and psychological barrier, particularly in countries where covering one's face is not a common habit.

Wearing a face mask, in fact, makes it hard to recognize if someone is smiling at you and to acknowledge non-verbal communication and emotions shared with facial expressions. This limitation has been noticed in the interactions with older, fragile, and cognitively impaired persons/patients, communication with whom strongly relies on body language (46). Not only in these contexts, but also in relation to day-to-day activities, especially with strangers, new communication skills are necessary, such as direct eye contact (47) and body gestures. Moreover, to communicate with those with hearing loss, special transparent masks have been proposed (48). As the fear of infection makes us more distrustful of strangers and even of friends and family members, to achieve the social interaction we were used to before the pandemic, a new demonstration of care and affection should be conceived.

“*When I walk and nobody is around me, I do not have my mask on the mouth and nose; however, when I'm approaching people, I pose it in the proper way and smile (with my eyes): I consider this a sort of ‘greetings and courtesy nod,' a way to say ‘I care for your health, do not be afraid by me, we will help each other.' I consider it as a message of solidarity.” [Lucia Martinelli, Italy]*

## Discussion

Although a “simple” face mask may not be considered in or of itself a sophisticated technological artifact, its systemic use in healthcare settings, its past adopted use in certain social contexts, and the current significant expansion of its application to public health measures (as evidenced through the testimonies and literature outlined above), it can be understood as a facet of a substantial technoscientific project. Importantly, face mask use in the case of COVID-19 has an obvious medical/healthcare connotation, even though face masks are used in many professions to protect the workers against inhaling dust or harmful substances. In fact, many mask types worn during the pandemic come from non-medical supplies (the standard “filtering face-piece” or FFP1 and FFP2 models). However, it is the medical-grade masks that serve as a reference point for all other (varieties of) face coverings.

Face mask wearing can be conceived within the practice of extending the medical science into the “outside world,” by making the behaviors and rituals of the society/culture more alike the scientific (laboratory) practices (25). The ideological repertoires used in doing so, however, depend critically on cultural differences among societies being thus transformed, and understanding them can help contextualize the political and social dimensions of implementing this public health measure. Such understanding can also serve as a resource for the introduction of other measures, as well as the uptake of face mask wearing in environments where it has not yet been adopted. In short, face masks are being recognized as boundary objects mediating between different individual and collective ideologies (31) and are as such artifacts with distinct politics (49).

The aim of this exploratory study was to understand face mask wearing in terms of public policies, individual behaviors and attitudes, and the collective experiences of the affected communities. The main results of our study highlight that the societal and personal practices of wearing (or not wearing) face masks are influenced by (1) individual perceptions of infection risk, (2) personal interpretations of responsibility and solidarity, (3) cultural traditions and religious imprinting, and (4) the need of expressing self-identity.

First, even for individuals who might not be concerned for their personal health and safety, the wearing of a face mask often indicates a level of care and respect toward others. The decision about wearing a face mask is mediated by standpoints on utility of face masks based on scientific knowledge and/or in the absence of scientific consensus also on political beliefs (17).

Second, the behaviors of others were described in the collected testimonies in terms of societal responsibilities and rituals of social interaction, highlighting the role of peers in shaping the individual behavior. The narratives shine a light on the perceived balance between protecting oneself and social responsibility, reasserting the notion “If the people wearing masks are protecting you, isn't it right that you should protect them in return?” (17). However, this leads to inherent contradictions in the behavioral change required. The interchangeability of *being at risk* and *being a risk* is particularly striking (34–36), making face mask wearing both an act of self-interest as well as altruism (42). In a similar vein, what could be perceived previously as anti-sociable behavior may now be beneficial for societal well-being (protection against the pandemic) and, in fact, preferred (3).

Third, our analysis highlighted that many countries, specifically those in Europe, that previously banned face coverings in public spaces are now mandating them. Face mask wearing has enjoyed varying levels of acceptance across different cultural, governmental, and religious environments; however, even in our study, we could show that the strict rules correspond to the better epidemiological situation (50). Moreover, the voluntary policy and insufficient compliance can be perceived as less fair allowing individuals to compromise epidemiological measures, while a mandatory policy appears as an effective, fair, and socially responsible (27). Although the mask can become a symbol of the fight against the virus or of neglect, it remains controversial who and when should have the control on the use of the symbol (51).

Fourth, the use of face masks preventing the spread of the virus is complemented or even upgraded by the use of face mask as a visual communication tool during times of lockdown and isolation providing a new way to communicate during a pandemic. This covers both political statements in relation to states' public health measures, as well as personal expression of raising awareness, collective solidarity, or just as a part of new pandemic-related esthetic.

We hope that this research will help develop new frameworks to guide a more holistic approach to understanding and enabling behavioral change among citizens, as well as enabling new models for non-verbal communication, noting specific challenges such as disability (46, 48). Recent articles highlight the need to develop new ways to communicate while wearing face masks through body language, particularly in terms of using eye contact to communicate emotion (52, 53). Also, there is an opportunity to develop new ethical frameworks to guide collective and individual decision making around face coverings. For health policy makers, our study highlights that public messaging plays a crucial role in institutional health communication and that in-depth knowledge of various cultures and ethics concerning health habits are relevant to informing and developing reliable information resources and policies for citizens during a global health pandemic.

However, this study was not without limitations. We acknowledge that our sample is yet representative of a group of intellectuals with a higher level of education, and therefore, the data cannot be generalized to the whole society. The methods we applied for data collection and analysis, however, fit the aim of our research: to explore the broad range of personal and social meanings of mask wearing in different countries. Furthermore, our sample combines the professional and personal observations by health and other experts providing a unique interdisciplinary perspective on face masks. Although we asked standard questions, we let people answer them in freestyle. We did not ask our authors to alter, explain, or correct their narratives in any way.

As shown by the narratives, during the COVID-19 crisis, inconsistent information may influence citizens' level of perceived risk, thus resulting in excessive fear or denial of the reality of the pandemic (54). The credibility and the source of the information may be crucial to promoting citizen compliance and best practice of face mask wearing. Here, the need to better communicate the complexities of (un)certainty (39) may be a useful lesson for public health officials and experts building “recommendation trust” in their advice (38).

From a purely medical perspective, the effectiveness of measures to contain the spread of the virus is independent of the geographic area where these measures are implemented. From a social scientific perspective, however, individual and public health is always embedded, in particular social, cultural, and political contexts. Because of these influencing factors, health measures and devices are imbued with particular meanings that differ across countries. The specific meaning of a device, such as a mask, acquires also shapes how people deal with it and how they integrate it (or not) into their everyday routines and practices (55). Ultimately, this implies that studying the personal and social meaning of mask wearing in different contexts is also necessary for the assessment of the effectiveness of face masks as a public health measure.

In conclusion, our study points out the need of an in-depth understanding of the various social, cultural, religious, and ethical considerations on health habits and attitudes in a time of pandemics. Additional knowledge about the variety of personal and collective understanding of face mask wearing is essential for designing more effective health communication during and beyond the COVID-19 pandemic.

## Data Availability Statement

The datasets presented in this study can be found in online repositories. The names of the repository/repositories and accession number(s) can be found below: http://dx.doi.org/10.17632/9s6fm7vdbc.1 (22).

## Ethics Statement

The studies involving human participants were reviewed and approved by Ethical Committees of the University of Edinburgh, Scotland, UK and the University of Zagreb, Faculty of Croatian Studies, Croatia. The patients/participants provided their written informed consent to participate in this study. Written informed consent was obtained from the individual(s) for the publication of any potentially identifiable images or data included in this article.

## Author Contributions

LM, VK, SG, CH, HM, NB, MP, and BP: designed the study. LM, VK, and SG: performed data acquisition, organization and analysis and wrote the first version of the manuscript. VK, MV, CH, HM, ZT, NB, MP, and BP: contributed to the interpretation of the results and critically revised manuscript. All authors approved the submission to the journal.

## Conflict of Interest

The authors declare that the research was conducted in the absence of any commercial or financial relationships that could be construed as a potential conflict of interest.
